# The Frequency and Predictive Factors of Change in Alcohol Consumption during the COVID-19 Pandemic: Results from a Multi-Country Longitudinal Study

**DOI:** 10.3390/nu16162591

**Published:** 2024-08-06

**Authors:** Carolien Verheij, Nadja Alexandrov, Erica I. Lubetkin, Gouke J. Bonsel, John N. Yfantopoulos, Mathieu F. Janssen, Stephanie C. E. Schuit, Suzanne Polinder, Pleunie P. M. Rood, Juanita A. Haagsma

**Affiliations:** 1 Department of Emergency Medicine, Erasmus MC—University Medical Center Rotterdam, 3000 CA Rotterdam, The Netherlands; p.rood@erasmusmc.nl (P.P.M.R.); j.haagsma@erasmusmc.nl (J.A.H.); 2 Department of Public Health, Erasmus MC—University Medical Center Rotterdam, 3000 CA Rotterdam, The Netherlands; 3 Department of Community Health and Social Medicine, CUNY School of Medicine, New York, NY 10031, USA; lubetkin@med.cuny.edu; 4 EuroQol Research Foundation, 3068 AV Rotterdam, The Netherlands; bonsel@euroqol.org; 5 MBA-Health Department of Economics, National and Kapodistrian University of Athens, 157 72 Athens, Greece; yfantopoulos@gmail.com; 6 Section Medical Psychology and Psychotherapy, Department of Psychiatry, Erasmus MC—University Medical Center Rotterdam, 3000 CA Rotterdam, The Netherlands; mf.bas.janssen@googlemail.com; 7 Department of Internal Medicine, Erasmus MC—University Medical Center Rotterdam, 3000 CA Rotterdam, The Netherlands; s.c.e.klein.nagelvoort@umcg.nl; 8 Board of Directors, University Medical Center Groningen, 3000 CA Rotterdam, The Netherlands

**Keywords:** alcohol, alcohol use disorder, COVID-19 pandemic, SARS-CoV-2, anxiety, depression, public health

## Abstract

Background: The COVID-19 pandemic has had multiple health and behavioral effects in the general population worldwide, including effects on nutritional and lifestyle behavior such as alcohol consumption. This study aimed to determine the frequency of and predictors for change in alcohol consumption two years after onset of the COVID-19 pandemic among participants from the general population of six countries. Methods: Longitudinal study design with 4999 participants (47% male; aged 18–75 years) from a general population cohort from six countries: Greece, Italy, the Netherlands, Sweden, the United Kingdom (UK) and the United States of America (US). Measurements: Three web-based surveys at different time waves: T1 = 22 April–1 June 2020; T2 = 2 May–29 June 2021 and T3 = 29 April–25 June 2022. The surveys included questions on self-reported retrospective alcohol consumption, demographics, health, anxiety and depression symptoms and recent life events. Results: Of 4999 respondents, most (82.3%) reported no change in drinking habits during the pandemic, whereas 12.5% reported drinking less and 5.1% drinking more. Predictive factors for increased alcohol consumption include age 35–54 years, male gender, high educational level, moderate-severe depression symptoms, excessive drinking before the COVID-19 pandemic, no change in general health status and job loss. Predictive factors for decreased alcohol consumption were age 18–34 years, male gender, having chronic disease(s), moderate-severe depression symptoms, excessive drinking before the pandemic and job loss. Conclusion: The proportion of participants who reported a decrease in alcohol consumption during the COVID-19 pandemic was higher compared to those who reported an increase. Excessive drinking before the pandemic, depression symptoms and job loss were predictors for both drinking more and drinking less alcohol during the COVID-19 pandemic with an stronger association for an increase in alcohol consumption.

## 1. Introduction

Since the start of 2019, globally, the COVID-19 pandemic has had an impact on multiple levels in daily life. Besides the burden of COVID-19 infection itself, multiple health and behavioral effects have been observed among non-affected persons, such as changes in nutritional and lifestyle behavior, including changes in alcohol consumption [1,2,3,4]. With regard to alcohol consumption, contradictory findings were observed. Some studies showed unchanged alcohol consumption, others showed a change, either decrease or increase [5,6,7,8,9,10]. These contradictory findings may, at least in part, be explained by the varying compositions of the different study populations [6,7,8,11,12,13]. Large general population studies that collected data from the first year of the COVID-19 pandemic (year 2020) showed an overall decrease in alcohol consumption, together with an increase in certain sub-groups [14,15,16].

In particular, persons with a pre-pandemic high-risk alcohol intake were more likely to increase their alcohol consumption further during the pandemic [15]. Another sub-group with a higher likelihood of increased alcohol consumption during the pandemic were those who initially experienced anxiety and depression symptoms. Several studies demonstrated significant associations between increased alcohol consumption and the onset or aggravation of depressive symptoms and/or anxiety symptoms [16,17,18,19,20,21,22]. This was also shown by Acuff et al. (2023), who demonstrated that depression and anxiety were associated with a greater likelihood of increased alcohol consumption during the first year of the COVID-19 pandemic [23].

Few studies investigated alcohol consumption more than one year after the onset of the COVID-19 pandemic and as a result, little is known about the persistence of this change in alcohol consumption during the later stages of the pandemic when governments gradually relaxed measures against the spread of COVID-19 (e.g., quarantine) and vaccination against COVID-19 became available [24]. So far, scientific evidence mainly draws from studies in the first year of the pandemic and predominantly relies on a cross-sectional design, which does not capture dynamic aspects of alcohol consumption [5,10,23].

In addition, detailed information on sub-groups that have a higher risk of increased alcohol consumption, including those who were directly affected by the pandemic due to for instance job loss, is currently lacking. This information is crucial to devise and implement tailored post-pandemic interventions of increased or excessive alcohol use in the general population.

Therefore, the current study addresses long term changes in alcohol consumption during the COVID-19 pandemic among participants from the general population of six countries, with a longitudinal dataset covering two years from the onset of the pandemic. Additionally, this study aimed to determine predictive factors for change in alcohol consumption, including socio-demographics, health and pre-existing alcohol consumption. These determinants may differ for increasing versus decreasing consumption.

## 2. Material and Methods

### 2.1. Study Design 

This study is part of the POPulation health impact of the CORoNavirus disease 2019 (COVID-19) pandemic (POPCORN) study. The POPCORN study employs a longitudinal study design to general population cohort from six countries: Greece, Italy, the Netherlands, Sweden, the United Kingdom (UK) and the United States of America (USA) [25,26]. Ethical approval was obtained from the Erasmus MC ethics review board (approval MEC-2020-0266; date 3 April 2020).

### 2.2. Data Collection and Consent

For the first wave of data collection (T1; 22 April–1 June 2020), an international market research agency recruited a representative sample of the participants among their pre-existing voluntary internet panels. The market research agency invited members from existing panels consisting of members of the general population residing in the selected countries to complete the online survey. Inclusion criteria were as follows: member of existing market research agency panel, aged 18–75 years at T1 and sufficient command of the dominant language in the respective countries. Exclusion criteria were as follows: aged <18 years or >75 years. Information on age, gender and the highest level of education was already known by the market research company. Based on this information, panelists were invited to fill out the questionnaire to ensure national representativeness across age, gender and highest attained level of education.

The panel members had given informed consent upon registration for the internet panel. Data were collected using online surveys. Participants who completed the first survey were contacted again by the market research agency to complete the second (T2; 2 May–29 June 2021) and third (T3; 29 April–25 June 2022) survey. Once participation of the survey had begun, it was not possible to skip questions or leave questions unanswered. The participants could leave the survey at any time. After completion of the survey, the participants received a monetary incentive or points from the market research company. The T1, T2 and T3 surveys were translated into the main official language of each country using translation software and subsequently translated back into English, except when validated translated versions of the instruments were available. Bilingual native speakers verified the translations independently. The survey data were pseudonymized.

#### 2.2.1. Alcohol Consumption

The T3 survey included questions about the average number of alcoholic beverages used per week before the pandemic (recall) and during the pandemic. The response options to these questions were “0–7 alcoholic beverages”, “8–14 alcoholic beverages”, “15–21 alcoholic beverages”, “22–28 alcoholic beverages” and “29 or more alcoholic beverages”. 

This information was used to identify respondents who met the criteria for excessive drinking before and during the COVID-19 pandemic. 

Excessive/heavy drinking was defined as drinking 8 or more (woman) or 15 or more (men) drinks per week [27,28]. Further, a question on excessive/heavy drinking at least once a week, during the pandemic was included during T3. This was defined as more than 3 (for woman) or more than 5 (for men) alcoholic consumptions, at least once a week, according to the Netherlands Institute of Mental Health and Addiction [29].

#### 2.2.2. Primary Outcome Measure

The primary outcome measure of this study was self-reported change in alcohol consumption during the COVID-19 pandemic. The T3 survey included a question that asked, “Have your drinking habits changed since the start of the pandemic?”. The response options to this question were “No”, “Yes, I stopped drinking alcohol”, “Yes, I drink less alcohol”, and “Yes, I drink more alcohol”. The response categories were recoded into “No change in alcohol consumption”, “Decrease in alcohol consumption” and “Increase in alcohol consumption”.

#### 2.2.3. Socio-Demographics and Health Characteristics

At T1, information about demographic characteristics (gender, age, highest level of education and occupational status) was collected. The highest level of education achieved was categorized into three groups according to the International Standard Classification of Education (ISCED) 2011: ISCED 0–2 (“Low”), ISCED 3–4 (“Middle”) and ISCED 5–8 (“High”). Information on occupational status, general health, chronic disease and COVID-19 infection was collected at T1, T2 and T3. Response options for occupational status were “In work: employee”, “In work: self-employed”, “Out of work for more than 1 year”, “Out of work for less than 1 year”, “Looking after others (e.g., a carer)”, “Student”, “Retired” and “Unable to work”. We recoded this variable into “In work”, “Out of work”, “Retired”, “Unable to work” and “Other”.

At T1, T2, T3 a self-report general health question was offered with the response options “very good”, “good”, “fair”, “bad” or “very bad”. Chronic disease status was measured by the presence of up to 11 chronic conditions (asthma or chronic bronchitis, heart disease, stroke, diabetes, arthritis, severe back complaints, arthrosis, cancer, memory problems, depression or anxiety disorder and/or other problems). The number of chronic diseases was categorized into two groups: “Zero” and “One or more”. Information about the presence of a (previous) COVID-19 infection during the pandemic was collected (“yes”/”no”).

In addition, the T1, T2 and T3 survey included the Patient Health Questionnaire-9 (PHQ-9) and the Generalized Anxiety Disorder Questionnaire (GAD-7) to measure symptoms of depression and anxiety, respectively. The PHQ-9 consists of nine items that measure the prevalence of depression-related symptoms in the past two weeks. The response options range from “Not at all” (“0”) to “Nearly every day” (“3”). The PHQ-9 sum score is the sum of the scores of each item and ranges from 0 to 27. In this study, the following interpretation is used for the PHQ-9 sum score (score; depression severity): 0–9 = None-mild and 10–27 = Moderate-severe [30].

The GAD-7 consists of seven items that ask the prevalence of anxiety-related symptoms in the past two weeks. The response options range from “Not at all” (“0”) to “Nearly every day” (“3”). The GAD-7 sum score is the sum of the scores of all items and ranges from 0 to 21. In this study, for the GAD-7 sum score the following interpretation is used (score; anxiety severity): 0–9 = None-mild and 10–27 = Moderate-severe [31].

#### 2.2.4. Recent Life Events Related to Health and Work Status

Recent life events “Change in general health” was captured by recording the difference in responses between wave 1 and wave 2. Change in general health was categorized into three groups: “No change”, “Improved at T2” and “Worsened at T2”. Job loss due to COVID-19 was assessed with a separate question in the T3 survey and had response options “yes” and “no”.

### 2.3. Statistical Analyses

The data were analyzed using SPSS (IBM Corp. 2017. IBM SPSS Statistics for Windows, Version 25.0. Armonk, NY: IBM Corp.). To test for a difference in respondent characteristics across the non-responders and the responders at T3, we used one-way ANOVA (for the continuous variable age) and Fisher’s exact and chi-square tests (for remaining categorical variables). For the T3 respondents demographic and health characteristics, the number of alcoholic beverages consumed per week before and during the pandemic, occurrence of recent life events and change in drinking behavior and excessive drinking were described using descriptive statistics. We tested for differences in demographic and health characteristics, number of alcoholic beverages consumed per week and occurrence of recent life events between the change in alcohol consumption for the three groups (“no change” versus “decrease in alcohol consumption” versus “increase in alcohol consumption”) using Kruskal–Wallis H test (continuous variables) and chi-square tests (categorical variables).

We used Sankey plots to explore patterns of change in the number of alcoholic beverages consumed per week before and during the COVID-19 pandemic. We used the Wilcoxon Signed-Rank Test to test for differences in number of alcoholic beverages consumed per week before and during the pandemic.

Next, we performed multinomial logistic regression analysis to investigate whether demographic variables (age, gender, country of residency, educational level, occupational status), health variables (presence chronic conditions, moderate to severe depression symptoms (PHQ-9 sum score ≥10) and moderate to severe anxiety symptoms (GAD-7 sum score ≥10), excessive drinking at onset of the COVID-19 pandemic and occurrence of life events (change in general health and job loss) were associated with a decrease or increase in alcohol consumption during the COVID-19 pandemic (reference category: no change). For all categorical variables included in the multinomial logistic regression analysis, we chose the largest category as the reference category. The gender variable category “other” was very small. We therefore choose to dichotomize the gender variable into males versus females and excluded respondents who reported “other” from the logistic regression analysis. We tested for the multicollinearity of the variables, and the goodness of fit of the model was determined with the Nagelkerke R-square test. A *p*-value below 0.05 was considered statistically significant.

## 3. Results

### 3.1. Study Sample

At T1 (April–May 2020), 19,902 respondents from Greece, Italy, the Netherlands, Sweden, the United Kingdom and the US completed the questionnaire. Of these, 4999 (response rate: 25%) also completed the questionnaire at T2 and T3. The comparison between the groups who completed all questionnaires and who completed only the first questionnaire are available in the Supplementary Table S1. The participants that completed all questionnaires were significantly older and more likely had a middle education level and no chronic disease compared to those who only completed the first questionnaire.

Table 1 shows the characteristics at T2 among the 4999 respondents in total. At T1, the median (IQR) age of all respondents was 53.0 (22). Slightly more than half of all respondents were female (52.6%), high-educated (50.7%) and employed (53.4%). Three out of four respondents had very good to good general health (73.4%) and 56.5% had no chronic condition(s). Approximately one in seven respondents had moderate to severe anxiety (14.7%) or depression symptoms (14.3%). Most participants (84.4%) reported drinking 0–7 alcoholic beverages per week before the COVID-19 pandemic and 2.6% met the criteria of excessive drinking before the pandemic. At T3, 3.0% reported excessive drinking and 19.3% reported heavy drinking at least once a week during the pandemic. In total, 4.4% lost their job during the pandemic and 13.0% experienced an improvement of general health, whereas 12.3% experienced worsened general health.

### 3.2. Change in Alcohol Consumption during the COVID-19 Pandemic

**Figure 1:**
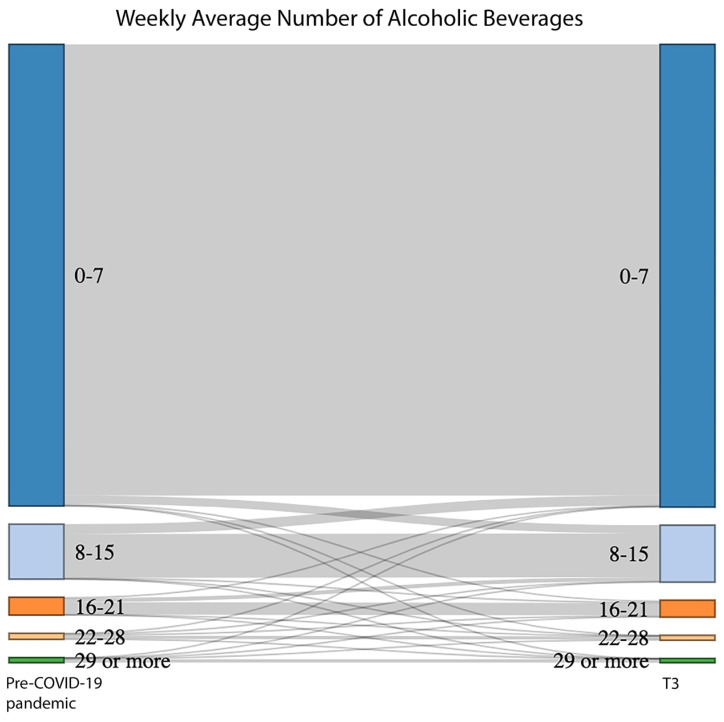
Sankey plot depicting the flow of change in average weekly alcohol consumption from before compared to during the COVID-19 pandemic (T3). The percentages for each category on the left and right side of the figure can be found in Table 1.

During the pandemic, most (82.3%) participants reported that their drinking habits did not change. One in eight (12.6%) respondents indicated that they decreased their alcohol consumption: 2.5% stopped drinking alcohol and 10.0% drink less alcohol. One in twenty (5.1%) respondents indicated that they drink more alcohol. Figure 1 shows the change in alcohol consumption from before compared to during the COVID-19 pandemic. Comparison of the distribution of number of alcoholic beverages consumed per week before and during the COVID-19 pandemic showed a significant difference (Z = −2.7, *p* = 0.006).

Respondents who stopped drinking or drink less alcohol were younger (48.0 vs. 52.5 years), lower educated (7.8% vs. 10.5% with high educational level), more often in work (57.6% vs. 52.1%), more often having one or more chronic disease (48.2% vs. 42.4%), moderate to severe depression (22.1% vs. 12.3%) or anxiety symptoms (22.3% vs. 12.5%) and more often drank 8 or more alcoholic beverages per week (28.3% vs. 11.9%) compared to those with no change in alcohol consumption.

Compared to those with no change in alcohol consumption, respondents who increased their alcohol consumption were younger (48.9 vs. 52.5), more often low educated (61.2% vs. 49.7%), more often in work (63.5% vs. 53.4%) or unable to work (7.1% vs. 4.6%), more often reported bad or very bad health (5.1% and 1.6% vs. 4.2% and 0.7%), more often suffered from chronic conditions (49.4% vs. 42.4%), more often had depression symptoms (27.5% vs. 12.3%) and anxiety symptoms (31.0% vs. 12.5%) and more often drank more than 8 alcoholic beverages per week before the pandemic (36.1% vs. 11.8%).

### 3.3. Predictive Factors for Change in Alcohol Consumption during the COVID-19 Pandemic

The results of the multinomial logistic regression analyses are shown in Table 2. The model had a good fit (Deviance: chi-square 345.5, *p* < 0.001; Nagelkerke Pseudo R-square = 0.098). A significantly higher odds of decrease in alcohol consumption during the COVID-19 pandemic was observed among those aged 18–34 years, males, residents of Greece, Italy, Sweden and the United Kingdom, those with a chronic disease, respondents with moderate to severe depression symptoms, respondents who drank excessively before the pandemic and respondents who lost their job due to COVID-19.

A significantly higher odds of increase in alcohol consumption during the COVID-19 pandemic was observed among those aged 35–54 years, males, respondents with a high education, respondents with moderate to severe depression symptoms, respondents who drank excessively before the pandemic, respondents who had no change in general health status and respondents who lost their job due to COVID-19. Results of the multinomial logistic regression analysis stratified by country are included in the Supplementary Materials (Supplementary Tables S2–S6). The models for Greece were not convergent due to a small sample size.

## 4. Discussion

This study determined frequency of and predictors for change in alcohol consumption two years after onset of the COVID-19 pandemic among participants from the general population of six countries. Before the pandemic, 2.6% of the participants met the criteria for excessive alcohol use. Definitions of excessive alcohol use differ among the literature. The cut-off points of eight or more (woman) or fifteen or more (men) drinks per week are the most common used definitions [27,28]. For drinks ‘at least once a week’, sometimes cut-offs for men of five or more drinks are used instead of more than five. In our questionnaire, the cut-off once a week for man of more than five (according to the Netherlands Institute of Mental Health and Addiction) was used. In general, the rate found in this study seems to be lower than in the general population [29,32]. During the COVID-19 pandemic this rate changed to 3%. One in twenty respondents reported heavy drinking at least once a week during the pandemic.

We found that one in eight out of 4999 respondents reported a decrease in alcohol consumption during the COVID-19 pandemic, whereas one in twenty reported an increase in alcohol consumption. It is important to note that an increase in self-reported alcohol consumption is not equivalent to clinically significant problems, although it is known that in general, an increase in alcohol use, even small amounts, brings higher risks of certain health problems [33].

Previous studies have shown different results; no changes, increases and decreases were all reported [5,6,7,8]. The results of this study are in line with previous larger (international) studies, where overall a decrease in alcohol consumption is seen, although some subpopulations show an increase in their alcohol intake during the pandemic [14,15,16]. There are studies that found a much higher increase in alcohol consumption during the COVID-19 pandemic [16,34]. For example, a longitudinal study by Barbosa et al. (2023) showed an increase in self-reported alcohol consumption and risky drinking patterns during the first year of the pandemic in the US [34]. This contradictory finding may be a result of a smaller window of data collection as well as a different sample population, as more studies have reported an increase in alcohol consumption in the US [8,23]. The difference in findings may also be explained by the difference in stringency of the lockdown measures against the spread of COVID-19 during the data collection periods.

Independent predictive factors for increased alcohol consumption during the COVID-19 pandemic were age 35–54 years old, male gender, high educational level, a PHQ-9 score of 10 or higher, excessive drinking before the COVID-19 pandemic and no change in general health status and job loss due to COVID-19. Independent predictive factors for decreased alcohol consumption during the COVID-19 pandemic were age 18–34 years old, male gender, one or more chronic disease(s), a PHQ-9 score of 10 or higher and excessive drinking before the pandemic and job loss due to COVID-19. Notably, four risk factors were associated with both an increase and a decrease in alcohol consumption (excessive drinking, PHQ-score of 10 or higher, job loss due to COVID-19 and male gender); however, these risk factors were more strongly associated with an increase in alcohol consumption during the pandemic.

Previous studies have reported that factors associated with an increase in drinking behavior include symptoms of depression and anxiety [16,23]. This is not fully in line with our findings; we found that depression symptoms were associated with both increase and decrease in alcohol consumption, but no association was found for moderate to severe anxiety symptoms. This may be because most previous studies that investigated the relation between change in alcohol consumption and anxiety and depression symptoms were cross-sectional studies, whereas in our study we measured anxiety and depression symptoms at the onset of the pandemic and their relationship with change in alcohol use at later stages in the pandemic. The results in the literature regarding age as a risk factor for changes in alcohol consumption during the pandemic are varying, possibly due to different populations and/or countries studied [23]. Pre-existing high drinking levels were found to be a risk factor for both an increase and decrease in alcohol use, with a stronger association for increase. A recent review by Acuff et al. (2023) also showed that up to half of all studies found a positive relationship between increases in consumption and pre-COVID alcohol use severity [23]. Job loss due to COVID-19 was found in this study to be both predictive for an increase and decrease in alcohol consumption, although the OR for an increase were higher compared to the OR for an decrease in alcohol use. The relation between job loss and an increase in alcohol consumption is in line with the findings of previous studies [23].

Analyses showed that residents of Greece, Italy, Sweden and the United Kingdom had significantly higher odds to decrease alcohol consumption during the COVID-19 pandemic compared to residents of the US. This study did not address cultural and political differences, differences in alcohol policy and and/or governmental restrictions between countries that may underly the differences in change in alcohol consumption patterns across countries that were observed in this study. Further research on that topic is warranted to gain more insight in factors that contribute to these cross-country differences in alcohol consumption patterns during a pandemic.

### Strengths and Limitations

Strengths of this study are that it is a multi-country, large cohort study and that information on several potential risk factors for change in alcohol consumption was collected over a two-year period. This allowed us to investigate a range of determinants related to socio-demographics, (mental) health, alcohol consumption and recent life events and their relationship with change in alcohol consumption during the COVID-19 pandemic. The data collection of the POPCORN study commenced in the first phase of the COVID-19 pandemic (April–June 2020), which means that information on anxiety and depression symptoms during this first phase of the pandemic could be included in this study. Studies have shown that anxiety and depression symptoms in the general population were highest in this first phase of the pandemic compared to later phases of the pandemic [35,36]. Because of the availability of anxiety and depression symptoms in this first phase of the pandemic, we could study the relationship between anxiety and depression symptoms at the beginning of the COVID-19 pandemic on change in alcohol consumption at later stages of the pandemic.

A limitation of this study is that the response to the T3 survey was rather low and that those who were younger, those with a low or high education level and those with one or more chronic disease were less likely to respond to all three surveys. This may have affected our findings with regard to the frequency of persons who reported a change in alcohol consumption, since younger age was associated with a change in alcohol use.

A second limitation is the possibility of social desirability bias, which is a commonly reported form of bias that often arises in surveys. It has been reported that people tend to under-report alcohol consumption and that people tend to be motivated to manage others’ impression of them [37,38]. It has been reported that respondents underreport their alcohol consumption by 20 to 33% [37]. Furthermore, selection bias in surveys may lead to the under-sampling of people with extreme levels of alcohol intake (e.g., students, homeless people and people with alcohol dependencies) [38]. Also, the subjects in this study were recruited from voluntary internet panels; therefore, sampling bias could have been introduced since it is possible that people with problematic drinking patterns are less willing to participate in a study that includes answering questions about alcohol use. These limitations could explain why the rates of excessive alcohol use in the study population were lower than expected compared to other reports on excessive alcohol use in the general population. Moreover, since we used recall in the reporting of alcohol consumption, recall bias is another limitation of this study that may present a threat to the internal validity. It has been observed that reported alcohol use decreases with increasing recall period [39,40].

Another limitation is that our study showed that country of residence is significantly associated with the change in alcohol consumption during the pandemic; however, our study design does not allow to investigate which country-level factors (e.g., government stringency level of measures against spread of COVID-19 (including lockdowns) or alcohol control policies during the pandemic) contributed to this association or could have been potential confounding factors in this association.

## 5. Conclusions

The majority of respondents reported no change in alcohol consumption. For those who did report change, the proportion of participants who reported a decrease in alcohol consumption was higher compared to those who reported an increase. Independent risk factors for an increase in alcohol use were excessive drinking before the pandemic, educational level, age, depression symptoms at the onset of the pandemic, no change in general health status and job loss due to COVID-19. Independent risk factors for a decrease in alcohol consumption were excessive drinking before the pandemic, age, depression symptoms at the onset of the COVID-19 pandemic, having one or more chronic diseases and job loss. Detailed information on long term changes in drinking patterns during the COVID-19 pandemic and the identification of sub-groups that were vulnerable to increased alcohol consumption is important to devise and implement tailored post-pandemic interventions of excessive alcohol use in the general population. Further research is warranted to investigate the prolonged impact of the COVID-19 pandemic on alcohol consumption. Also, further research is warranted to investigate different approaches and strategies within the population at risk. Given that mental health symptoms were associated with increase in alcohol consumption, it is key to provide robust mental health support, including accessible counseling services, mental health hotlines and community-based mental health programs as well as integrate routine mental health screenings into primary care visits, especially for individuals with a history of excessive drinking. In addition, we recommend to implement policies that regulate availability of alcohol during a pandemics and social distancing and quarantine measures.

## Figures and Tables

**Table 1:** Characteristics of respondents, total and by change in alcohol consumption category.

		Total	Change in Alcohol Consumption ^2^			*p* Value
			No change	Decrease	Increase	
n		4999 (100%)	4116 (82.3%)	628 (12.6%)	255 (5.1%)	
Variable	Categories	Frequency (% of variable)				
**Demographic characteristics**						
Age ^1^	Median (IQR)	53.0 (22)	54.0 (21)	48.0 (24)	49.0 (20)	<0.001
	Mean (SD)	51.8 (13.6)	52.5 (13.4)	48.0 (14.2)	48.9 (13.1)	
Age category ^1^	18–34	646 (12.9)	482 (11.7)	130 (20.7)	43 (13.3)	<0.001
	35–54	2028 (40.6)	1627 (39.5)	127 (43.2)	130 (51.0)	
	55–74	2325 (46.5)	2007 (48.8)	227 (36.1)	91 (35.7)	
Gender ^1^	Male	2369 (47.4)	1928 (46.8)	304 (48.4)	137 (53.7)	0.19
	Female	2627 (52.6)	2186 (53.1)	323 (51.4)	118 (46.3)	
	Other	3 (0.1)	2 (0.05)	1 (0.2)	0 (0)	
Country	Greece	376 (7.5)	292 (7.1)	69 (11.0)	15 (27.1)	<0.001
	Italy	1165 (23.3)	929 (22.6)	183 (29.1)	53 (5.9)	
	Netherlands	644 (12.9)	571 (13.9)	54 (8.6)	19 (20.8)	
	Sweden	729 (14.6)	604 (14.7)	96 (15.3)	29 (7.5)	
	UK	873 (17.5)	674 (16.4)	130 (20.7)	69 (11.4)	
	US	1212 (24.2)	1046 (25.4)	96 (15.3)	70 (27.5)	
Education level ^1^	Low	492 (9.8)	2047 (49.7)	331 (52.7)	156 (61.2)	<0.001
	Middle	1973 (39.5)	1636 (39.7)	248 (39.5)	89 (34.9)	
	High	2534 (50.7)	433 (10.5)	49 (7.8)	10 (3.9)	
Occupational status ^1^	In work	2670 (53.4)	2146 (52.1)	362 (57.6)	162 (63.5)	<0.001
	Out of work	554 (11.1)	451 (11.0)	78 (12.4)	25 (9.8)	
	Retired	1236 (26.4)	1087 (26.4)	108 (17.2)	41 (16.1)	
	Unable to work	238 (4.6)	191 (4.6)	29 (4.6)	18 (7.1)	
	Other	241 (5.9)	241 (5.9)	51 (8.1)	9 (3.5)	
Living situation ^1^	Living alone	1054 (21.1)	873 (21.7)	132 (21.0)	49 (19.2)	0.75
**Health characteristics**						
General health ^1^	Very good	929 (18.6)	791 (19.2)	99 (15.8)	39 (15.3)	<0.05
	Good	2731 (54.8)	2234 (54.3)	366 (58.3)	131 (51.4)	
	Fair	1103 (21.1)	892 (21.7)	143 (22.8)	68 (26.7)	
	Bad	200 (4.0)	171 (4.2)	16 (2.5)	13 (5.1)	
	Very bad	36 (0.7)	28 (0.7)	4 (0.6)	4 (1.6)	
Chronic condition ^1^	One or more	2174 (43.5)	1745 (42.4)	303 (48.2)	126 (49.4)	<0.05
Depression symptoms ^1^	PHQ-9 ≥ 10	716 (14.3)	507 (12.3)	139 (22.1)	70 (27.5)	<0.001
Anxiety symptoms^1^	GAD-7 ≥ 10	735 (14.7)	516 (12.5)	140 (22.3)	79 (31.0)	<0.001
**Alcohol consumption (per week)**						
Before the pandemic (recall) ^2^	0–7	4241 (84.4)	3628 (88.1)	450 (71.7)	163 (63.9)	<0.001
	8–14	521 (10.4)	346 (8.4)	120 (19.1)	55 (21.6)	
	15–21	154 (3.1)	91 (2.2)	42 (6.7)	21 (8.2)	
	22–28	45 (0.9)	25 (0.6)	9 (1.4)	11 (4.3)	
	29 or more	38 (0.8)	26 (0.6)	7 (1.1)	5 (2.0)	
Excessive drinking before the pandemic ^2$^	Yes	128 (2.6)	69 (1.7)	35 (5.6)	24 (9.4)	<0.001
During the pandemic (T3) ^2^	0–7	4231 (84.6)	3628 (88.1)	519 (82.9)	84 (32.9)	<0.001
	8–14	504 (10.1)	346 (8.4)	66 (10.5)	92 (36.1)	
	15–21	162 (3.2)	91 (2.2)	28 (4.5)	43 (16.9)	
	22–28	56 (1.1)	25 (0.6)	11 (1.8)	20 (7.8)	
	29 or more	46 (0.9)	26 (0.6)	4 (0.6)	16 (6.3)	
Excessive during the pandemic ^2$^	Yes	149 (3.0)	69 (1.7)	29 (4.6)	51 (20.0)	<0.001
**Recent life events**						
Job loss due to COVID-19 pandemic ^2^	Yes	222 (4.4)	138 (3.4)	57 (9.1)	27 (10.6)	<0.001
Change in health status ^3^	No change	3731 (74.6)	3047 (74.0)	483 (76.9)	201 (78.8)	0.07
	Improved	652 (13.0)	561 (13.6)	70 (11.1)	21 (8.2)	
	Worsened	616 (12.3)	508 (12.3)	75 (11.9)	33 (12.9)	

^1^ = Variable data are based on data retrieved at T1 POPCORN questionnaire (April–May 2020). ^2^ = Variable data are based on data retrieved at T3 POPCORN questionnaire (May–June 2022). ^3^ = Variable data are based on data retrieved with the T1 and T2 POPCORN questionnaire (May–June 2021). ^$^ = Criteria for excessive drinking are dependent on gender (male/female), therefore excessive drinking was only assessed for those with gender male or female.

**Table 2:** Multivariate multinomial logistic regression analyses for change in alcohol consumption (n = 4996 #). Dependent variable category ‘No change’ is the reference variable.

		Decrease in Alcohol Consumption				Increase in Alcohol Consumption			
Variables	Categories	OR	5%	95%	*p* Value	OR	5%	95%	*p* Value
**Age category**	18–34	1.65	1.24	2.21	0.001	1.18	0.73	1.90	0.49
	35–54	1.18	0.94	1.49	0.15	1.43	1.02	2.01	0.04
	55–74 (ref)								
**Gender**	Male	1.20	1.00	1.43	0.048	1.48	1.13	1.93	0.004
	Female (ref)								
**Country**	Greece	2.20	1.55	3.12	<0.001	0.70	0.39	1.27	0.24
	Italy	1.97	1.49	2.60	<0.001	0.80	0.54	1.19	0.28
	Netherlands	1.08	0.76	1.56	0.66	0.61	0.36	1.05	0.08
	Sweden	1.66	1.22	2.28	0.001	0.76	0.48	1.21	0.24
	UK	1.91	1.43	2.55	<0.001	1.26	0.88	1.81	0.21
	US (ref)								
**Education level**	Low	0.81	0.57	1.14	0.22	0.41	0.21	0.82	0.01
	Middle	0.95	0.79	1.14	0.57	0.77	0.58	1.02	0.07
	High (ref)								
**Occupational status**	Out of work	0.85	0.64	1.14	0.28	0.72	0.45	1.14	0.16
	Retired	0.78	0.59	1.03	0.08	0.70	0.46	1.07	0.10
	Unable to work	0.92	0.59	1.43	0.71	1.05	0.59	1.87	0.86
	Other	1.18	0.84	1.67	0.34	0.60	0.29	1.21	0.15
	In work (ref)								
**Living situation**	Living alone	1.11	0.89	1.38	0.35	0.87	0.62	1.22	0.43
	Not living alone (ref)								
**General health**	Fair to very bad	0.87	0.70	1.08	0.21	1.17	0.86	1.60	0.33
	Very good to good (ref)								
**Chronic condition ^1^**	One or more	1.32	1.09	1.60	0.004	1.17	0.87	1.56	0.30
	None (ref)								
**Depression symptoms**	PHQ-9 ^1^ ≥10	1.35	1.01	1.80	0.04	2.24	1.49	3.36	<0.001
	PHQ-9 ^1^ <10 (ref)								
**Anxiety symptoms**	GAD-7 ^2^ ≥10	1.27	0.94	1.71	0.12	1.27	0.82	1.96	0.28
	GAD-7 ^2^ <10 (ref)								
**Excessive drinking before the pandemic**	Yes	3.43	2.22	5.28	<0.001	5.30	3.19	8.79	<0.001
	No (ref)								
**Job loss**	Yes	2.33	1.66	3.27	<0.001	2.80	1.77	4.44	<0.001
	No (ref)								
**Change in health status**	Improved	0.82	0.62	1.08	0.15	0.56	0.35	0.89	0.02
	Worsened	0.97	0.74	1.27	0.84	0.95	0.64	1.41	0.81
	No change (ref)								

Deviance: chi-square 345.5, *p* < 0.001; Nagelkerke Pseudo R-square = 0.098. # Respondents who reported “other” as gender (n = 3) were excluded from the logistic regression analysis. ^1^ Patient Health Questionnaire-9. ^2^ Generalized Anxiety Disorder Questionnaire-7.

## Data Availability

Due to privacy restrictions data are available from the corresponding author, upon reasonable request.

## Supplementary Materials

The following supporting information can be downloaded at https://www.mdpi.com/article/10.3390/nu16162591/s1. Table S1. Non-response analysis of the dropout in 2020 (T1) vs respondents in 2022 (T3), at T1; Table S2. Multivariate multinomial logistic regression analyses for change in alcohol consumption (United Kingdom; n = 871#); Table S3. Multivariate multinomial logistic regression analyses for change in alcohol consumption (United States; n = 1212); Table S4. Multivariate multinomial logistic regression analyses for change in alcohol consumption (Sweden; n = 728#); Table S5. Multivariate multinomial logistic regression analyses for change in alcohol consumption (Netherlands; n = 644); Table S6. Multivariate multinomial logistic regression analyses for change in alcohol consumption (Italy; *n* = 1165); Paragraph that describes the characteristics of the countries included in this study.
