# Supplementary material for: The Frequency and Predictive Factors of Change in Alcohol Consumption during the COVID-19 Pandemic: Results from a Multi-Country Longitudinal Study
Source: Nutrients. 2024 Aug 6;16(16):2591. doi: 10.3390/nu16162591 (PMC11357368; doi:10.3390/nu16162591)
Supplement: Supplementary file 1 [file nutrients-16-02591-s001.zip › nutrients-3072522-supplementary.pdf]

SUPPLEMENTARY MATERIAL

The Frequency and Predictive Factors of Change in Alcohol Consumption during the COVID-19 Pandemic: Results from a Multi-Country Longitudinal Study

Carolien Verheij [1,*], Nadja Alexandrov [2], Erica I. Lubetkin [3], Gouke J. Bonsel [4], John N. Yfantopoulos [5], Mathieu F. Janssen [6], Stephanie C. E. Schuit [7,8], Suzanne Polinder [2], Pleunie P. M. Rood [1] and Juanita A. Haagsma [1,2]

***Correspondence:** Carolien Verheij c.verheij.1@erasmusmc.nl.

S1.1 Non-response analysis (T1 versus T3)

Table S1. Non-response analysis of the dropout in 2020 (T1) vs respondents in 2022 (T3), at T1

		Dropout after T1 (n=14903)	*Completer T1 and T2 (n=4999)*	*p-value*
Gender	Male	6925 (46.5)	2369 (47.4)	.009
	Female	7939 (53.3)	2627 (52.6)	
	Other	39 (0.3)	3 (0.1)	
Age	Median (IQR)	43 (26)	53 (22)	<.001
	Mean (SD)	44.2 (16)	51.8 (13.6)	
Age category	18 – 24	1814 (12.2)	132 (2.6)	<.001
	25 – 34	3065 (20.6)	514 (10.3)	
	35 – 44	3093 (20.8)	926 (18.5)	
	45 – 54	2618 (17.6)	1102 (22.0)	
	55 – 64	2124 (14.3)	1213 (24.3)	
	65 – 75	2189 (14.7)	1112 (22.2)	
Educational level	High	7853 (52.7)	2534 (50.7)	.002
	Middle	5478 (36.8)	1973 (39.5)	
	Low	1572 (10.5)	492 (9.8)	
Country	Greece	646 (4.3)	376 (7.5)	<.001
	Italy	2047 (13.7)	1165 (23.3)	
	The Netherlands	2652 (17.8)	644 (12.9)	
	Sweden	2490 (16.7)	729 (14.6)	
	United Kingdom	2361 (15.8)	873 (17.5)	
	United States	4707 (31.6)	1212 (24.2)	
Chronic condition(s)	None	7760 (52.1)	2825 (56.5)	<.001
	One or more	7143 (47.9)	2174 (43.5)	

S1.2 Multinomial regression analysis results by country

Table S2. Multivariate multinomial logistic regression analyses for change in alcohol consumption (United Kingdom; n = 871[#])

		Decrease in alcohol consumption				Increase in alcohol consumption			
Variables	Categories	OR	5%	95%	p-value	OR	5%	95%	p-value
Age category	18 – 34	1.02	0.52	2.00	0.96	1.05	0.42	2.65	0.92
	35 – 54	0.91	0.55	1.50	0.71	1.08	0.55	2.13	0.83
	55-74 (ref)								
Gender	Male	1.21	0.81	1.81	0.35	1.39	0.81	2.39	0.23
	Female (ref)								
Education level	Low	0.68	0.19	2.49	0.56	.	.	.	.
	Middle	0.89	0.60	1.33	0.58	0.73	0.42	1.26	0.26
	High (ref)								
Occupational status	Out of work	1.17	0.53	2.60	0.70	0.26	0.03	2.03	0.20
	Retired	0.84	0.48	1.49	0.56	0.78	0.35	1.72	0.54
	Unable to work	0.97	0.43	2.18	0.94	1.70	0.64	4.48	0.29
	Other	1.46	0.56	3.79	0.44	0.42	0.05	3.22	0.40
	In work (ref)								
Living situation	Living alone	1.34	0.85	2.12	0.20	0.66	0.32	1.34	0.25
	Not living alone (ref)								
General health	Fair to very bad	0.77	0.47	1.27	0.31	1.21	0.62	2.34	0.58
	Very good to good (ref)								
Chronic condition	One or more	1.26	0.81	1.96	0.30	0.74	0.40	1.37	0.34
	None (ref)								
Depression symptoms	PHQ-9≥10	0.87	0.45	1.71	0.69	1.72	0.73	4.02	0.21
	PHQ-9<10 (ref)								
Anxiety symptoms	GAD-7≥10	1.80	0.92	3.51	0.08	0.99	0.40	2.50	0.99
	GAD-7<10 (ref)								
Excessive drinking before the pandemic	Yes	3.07	1.48	6.36	0.003	5.78	2.63	12.71	<0.001
	No (ref)								
Job loss	Yes	1.52	0.57	4.05	0.40	2.69	0.89	8.11	0.08
	No (ref)								
Change in health status	Improved	0.72	0.37	1.42	0.35	0.24	0.06	1.02	0.053
	Worsened	1.44	0.84	2.46	0.18	1.06	0.49	2.26	0.89
	No change (ref)								

Deviance: Chi-square 64.6, p=0.002; Nagelkerke Pseudo R-square = 0.096.

[#] Respondents who reported "other" as gender (n=2) were excluded from the logistic regression analysis.

Table S3. Multivariate multinomial logistic regression analyses for change in alcohol consumption (United States; n = 1212[#])

		Decrease in alcohol consumption				Increase in alcohol consumption			
Variables	Categories	OR	5%	95%	p-value	OR	5%	95%	p-value
Age category	18 – 34	2.04	1.00	4.19	0.051	0.32	0.07	1.51	0.15
	35 – 54	1.19	0.69	2.06	0.53	1.85	1.01	3.40	0.046
	55-74 (ref)								
Gender	Male	0.89	0.57	1.39	0.62	1.10	0.66	1.84	0.71
	Female (ref)								
Education level	Low	1.95	0.62	6.15	0.26	1.49	0.45	4.94	0.51
	Middle	1.03	0.65	1.66	0.89	0.89	0.50	1.58	0.69
	High (ref)								
Occupational status	Out of work	1.26	0.63	2.50	0.52	0.96	0.40	2.31	0.93
	Retired	0.70	0.38	1.31	0.27	0.74	0.35	1.58	0.44
	Unable to work	0.44	0.12	1.64	0.22	2.06	0.77	5.50	0.15
	Other	0.90	0.34	2.39	0.84	.	.	.	
	In work (ref)								
Living situation	Living alone	0.86	0.50	1.48	0.59	0.66	0.34	1.30	0.24
	Not living alone (ref)								
General health	Fair to very bad	0.84	0.49	1.43	0.52	0.73	0.39	1.37	0.32
	Very good to good (ref)								
Chronic condition	One or more	2.11	1.35	3.30	0.001	1.18	0.69	2.02	0.55
	None (ref)								
Depression symptoms	PHQ-9≥10	1.20	0.52	2.78	0.66	4.32	1.99	9.39	<0.001
	PHQ-9<10 (ref)								
Anxiety symptoms	GAD-7≥10	1.31	0.53	3.22	0.56	0.81	0.32	2.01	0.65
	GAD-7<10 (ref)								
Excessive drinking before the pandemic	Yes	3.51	1.32	9.35	0.01	1.99	0.44	9.09	0.38
	No (ref)								
Job loss	Yes	2.81	1.35	5.82	0.006	3.08	1.32	7.21	0.009
	No (ref)								
Change in health status	Improved	1.12	0.62	2.02	0.71	0.87	0.40	1.85	0.71
	Worsened	0.57	0.26	1.23	0.15	1.02	0.50	2.07	0.97
	No change (ref)								

Deviance: Chi-square 96.2, p<0.001; Nagelkerke Pseudo R-square = 0.122.

[#] Respondents who reported "other" as gender (n=2) were excluded from the logistic regression analysis.

Table S4. Multivariate multinomial logistic regression analyses for change in alcohol consumption (Sweden; n = 728#)

		Decrease in alcohol consumption				Increase in alcohol consumption			
Variables	Categories	OR	5%	95%	p-value	OR	5%	95%	p-value
Age category	18 – 34	1.04	0.44	2.47	0.93	0.25	0.03	2.26	0.22
	35 – 54	1.41	0.81	2.48	0.23	0.93	0.36	2.41	0.89
	55-74 (ref)								
Gender	Male	1.00	0.63	1.59	0.99	2.57	1.10	6.03	0.03
	Female (ref)								
Education level	Low	0.71	0.28	1.82	0.48	0.35	0.04	2.92	0.33
	Middle	1.28	0.79	2.08	0.32	1.44	0.61	3.39	0.40
	High (ref)								
Occupational status	Out of work	0.57	0.25	1.32	0.19	0.83	0.24	2.90	0.77
	Retired	0.69	0.36	1.31	0.25	0.68	0.25	1.82	0.44
	Unable to work	0.82	0.31	2.18	0.70	.	.	.	.
	Other	1.82	0.62	5.32	0.28	.	.	.	.
	In work (ref)								
Living situation	Living alone	1.19	0.73	1.91	0.49	1.33	0.59	3.00	0.50
	Not living alone (ref)								
General health	Fair to very bad	0.82	0.46	1.47	0.50	2.18	0.87	5.44	0.10
	Very good to good (ref)								
Chronic condition	One or more	1.22	0.72	2.06	0.46	0.82	0.33	2.04	0.67
	None (ref)								
Depression symptoms	PHQ-9≥10	2.68	1.19	6.04	0.02	3.64	0.94	14.12	0.06
	PHQ-9<10 (ref)								
Anxiety symptoms	GAD-7≥10	1.18	0.54	2.55	0.68	1.10	0.29	4.22	0.89
	GAD-7<10 (ref)								
Excessive drinking before the pandemic	Yes	1.68	0.38	7.50	0.50	2.54	0.21	31.40	0.47
	No (ref)								
Job loss	Yes	2.18	0.87	5.48	0.10	3.37	0.80	14.25	0.10
	No (ref)								
Change in health status	Improved	0.52	0.26	1.06	0.07	0.25	0.05	1.13	0.07
	Worsened	0.96	0.53	1.77	0.91	0.54	0.15	1.95	0.35
	No change (ref)								

Deviance: Chi-square 71.5, p<0.001; Nagelkerke Pseudo R-square = 0.140. # Respondents who reported "other" as gender (n=1) were excluded from the logistic regression analysis.

Table S5. Multivariate multinomial logistic regression analyses for change in alcohol consumption (Netherlands; n = 644)

		Decrease in alcohol consumption				Increase in alcohol consumption			
Variables	Categories	OR	5%	95%	p-value	OR	5%	95%	p-value
Age category	18 – 34	2.79	0.89	8.77	0.08	0.85	0.13	5.46	0.86
	35 – 54	1.40	0.65	3.02	0.40	1.35	0.42	4.28	0.61
	55-74 (ref)								
Gender	Male	1.63	0.88	3.01	0.12	1.82	0.62	5.34	0.27
	Female (ref)								
Education level	Low	2.19	1.04	4.62	0.04	0.16	0.02	1.40	0.10
	Middle	1.04	0.48	2.24	0.92	0.75	0.25	2.24	0.61
	High (ref)								
Occupational status	Out of work	1.16	0.40	3.37	0.78	0.49	0.05	4.48	0.53
	Retired	1.01	0.44	2.31	0.99	0.19	0.02	1.76	0.15
	Unable to work	0.74	0.24	2.29	0.60	0.76	0.13	4.51	0.76
	Other	0.43	0.09	2.06	0.29	2.19	0.46	10.39	0.33
	In work (ref)								
Living situation	Living alone	1.10	0.58	2.09	0.78	1.54	0.52	4.60	0.44
	Not living alone (ref)								
General health	Fair to very bad	1.22	0.59	2.49	0.59	0.69	0.18	2.58	0.58
	Very good to good (ref)								
Chronic condition	One or more	1.22	0.62	2.43	0.56	1.30	0.40	4.21	0.66
	None (ref)								
Depression symptoms	PHQ-9≥10	2.43	0.69	8.56	0.17	2.03	0.40	10.32	0.39
	PHQ-9<10 (ref)								
Anxiety symptoms	GAD-7≥10	0.49	0.12	1.97	0.31	2.89	0.55	15.16	0.21
	GAD-7<10 (ref)								
Excessive drinking before the pandemic	Yes	6.55	2.12	20.28	0.001	5.00	0.72	34.80	0.10
	No (ref)								
Job loss	Yes	3.19	0.54	18.70	0.20	2.94	0.21	41.55	0.42
	No (ref)								
Change in health status	Improved	0.64	0.27	1.56	0.33	0.28	0.03	2.29	0.24
	Worsened	0.62	0.26	1.51	0.30	1.59	0.51	4.95	0.43
	No change (ref)								

Deviance: Chi-square 54.6, p=0.024; Nagelkerke Pseudo R-square = 0.143.

Table S6. Multivariate multinomial logistic regression analyses for change in alcohol consumption (Italy; n = 1165)

		Decrease in alcohol consumption				Increase in alcohol consumption			
Variables	Categories	OR	5%	95%	p-value	OR	5%	95%	p-value
Age category	18 – 34	1.79	1.03	3.09	0.04	3.55	1.16	10.83	0.03
	35 – 54	1.28	0.80	2.06	0.31	2.67	0.98	7.31	0.06
	55-74 (ref)								
Gender	Male	1.28	0.91	1.80	0.15	1.67	0.91	3.08	0.10
	Female (ref)								
Education level	Low	0.39	0.21	0.74	0.004	0.39	0.11	1.40	0.15
	Middle	0.70	0.49	1.01	0.054	0.61	0.32	1.16	0.13
	High (ref)								
Occupational status	Out of work	0.70	0.42	1.17	0.18	0.35	0.13	0.96	0.04
	Retired	0.72	0.37	1.40	0.33	0.36	0.07	1.92	0.23
	Unable to work	3.89	0.95	15.81	0.06	3.89	0.96	15.81	0.06
	Other	1.29	0.74	2.25	0.36	0.68	0.22	2.09	0.50
	In work (ref)								
Living situation	Living alone	0.79	0.42	1.47	0.454	1.19	0.43	3.27	0.73
	Not living alone (ref)								
General health	Fair to very bad	0.85	0.56	1.29	0.450	1.26	0.64	2.48	0.51
	Very good to good (ref)								
Chronic condition[1]	One or more	1.24	0.86	1.80	0.24	1.82	0.96	3.44	0.07
	None (ref)								
Depression symptoms	PHQ-9≥10	1.29	0.78	2.14	0.32	1.96	0.85	4.50	0.11
	PHQ-9<10 (ref)								
Anxiety symptoms	GAD-7≥10	1.16	0.68	1.99	0.58	1.66	0.72	3.84	0.24
	GAD-7<10 (ref)								
Excessive drinking before the pandemic	Yes	4.54	1.51	13.70	0.007	11.17	3.18	39.32	<0.001
	No (ref)								
Job loss	Yes	2.34	1.29	4.27	0.005	2.25	0.83	6.12	0.11
	No (ref)								
Change in health status	Improved	1.13	0.68	1.86	0.64	1.17	0.49	2.81	0.73
	Worsened	1.13	0.66	1.95	0.66	0.74	0.25	2.22	0.59
	No change (ref)								

Deviance: Chi-square 54.6, p=0.024; Nagelkerke Pseudo R-square = 0.143.

The multinomial logistic regression analysis could not be performed for Greece due to the low number of respondents residing in Greece (n=376).

S1.3 Characteristics of the countries included in this study

Economic development, alcohol policy and restrictions during COVID-19 pandemic

Greece

Number of Inhabitants: Greece has a population of approximately 10.4 million people as of 2023.
Economic Development: Gross domestic product was 217.6 billion United States Dollars (USD) in 2022. Greece faced a severe financial crisis starting in 2009, leading to a prolonged recession and austerity measures. However, Greece has shown signs of recovery in recent years, with GDP growth and improved employment rates.
Alcohol Policy:

- Legal drinking age is 18, and alcohol is widely available.
- There a few restrictions on advertising.
- There are regulations in place regarding drinking and driving.

COVID-19 Pandemic Measures: Greece implemented strict measures to control the spread of COVID-19, in particular during the first stages of the pandemic. These measures included nationwide lockdowns, travel restrictions, mandatory mask-wearing, and social distancing rules.

Italy

Number of Inhabitants: Italy has a population of approximately 59 million people as of 2023.
Economic Development: Gross domestic product was 2.05 trillion USD in 2022.
Alcohol Policy:

- Legal drinking age is 18, and alcohol is widely available.
- Drinking is often associated with meals and socializing, and there is a cultural emphasis on moderation.
- There are regulations on drinking and driving, and public campaigns to promote responsible drinking.

COVID-19 Pandemic Measures: Italy was one of the first European countries to be severely affected by the COVID-19 outbreak. The government implemented stringent measures, including nationwide lockdowns, travel bans, mandatory mask-wearing, and social distancing rules.

The Netherlands

Number of Inhabitants: The Netherlands has a population of approximately 17.8 million people as of 2023.
Economic Development: Gross domestic product was 1000.9 trillion USD in 2022.
Alcohol Policy:

- Legal drinking age is 18 for both purchasing and consuming alcohol.
- Alcohol is widely available.
- There are regulations on advertising and selling alcohol, especially to minors.
- There are high penalties for drinking and driving.

COVID-19 Pandemic Measures: During the COVID-19 pandemic, The Netherlands implemented a range of measures to control spread of COVID-19. These measures included lockdowns, social distancing mandates, travel restrictions, and mandatory mask-wearing in certain settings.

Sweden
Number of Inhabitants: Sweden has a population of approximately 10.5 million people as of 2023.
Economic Development: Gross domestic product was 591.2 billion in 2022. Sweden is characterized by a strong welfare state and high standards of living.
Alcohol Policy:

- Sweden has a strict alcohol policy compared to many other European countries.
- Legal drinking age is 18 for buying alcohol in bars and restaurants, but it is 20 for purchasing alcohol in government-run stores.
- Government controls the sale and distribution of alcohol to limit consumption and reduce alcohol-related harms.
- There are high taxes on alcohol.
- Advertising of alcohol is highly regulated.

COVID-19 Pandemic Measures: The approach that the Swedish government took during the COVID-19 pandemic was different from many other European countries. Initially the measures in Sweden were less strict compared to other countries, relying more on voluntary guidelines and recommendations rather than forced lockdowns. Measures included promoting social distancing, encouraging remote work, and recommending against large gatherings. Over time, Sweden implemented stricter measures, including limits on public gatherings and restrictions on businesses.

United Kingdom
Number of Inhabitants: The United Kingdom has a population of approximately 67 million people as of 2023.
Economic Development: Gross domestic product was 3.1 trillion in 2022. The UK economy has faced challenges due to Brexit in 2020.
Alcohol Policy:

- Legal drinking age is 18.
- Alcohol is widely available, and the country has a strong pub culture.
- There are regulations regarding the sale of alcohol, including licensing laws and restrictions on advertising and promotions.
- The UK also has laws against drinking and driving, with strict penalties for violations.

COVID-19 Pandemic Measures: The government of the UK implemented strict measures to limit the spread of COVID-19, including nationwide lockdowns, social distancing mandates, mask requirements, and travel restrictions.

United States
Number of Inhabitants: The United States has a population of approximately 331 million people as of 2023.
Economic Development: Gross domestic product was 25.4 trillion USD in 2022. The United States has the world's largest economy.
Alcohol Policy

- Legal drinking age in the United States is 21.
- Alcohol laws vary by state, but generally, there are restrictions on the sale and consumption of alcohol.
- Regulations include licensing requirements for sellers, restrictions on advertising, and laws against drinking and driving.

COVID-19 Pandemic Measures: In the United States the stringency and enforcement of measures that were implemented against the spread of COVID-19 differed across states.
